# MonoDCN: Monocular 3D object detection based on dynamic convolution

**DOI:** 10.1371/journal.pone.0275438

**Published:** 2022-10-04

**Authors:** Shenming Qu, Xinyu Yang, Yiming Gao, Shengbin Liang

**Affiliations:** School of Software, Henan University, Kaifeng, Henan, China; Wuhan University of Science and Technology, CHINA

## Abstract

3D object detection is vital in the environment perception of autonomous driving. The current monocular 3D object detection technology mainly uses RGB images and pseudo radar point clouds as input. The methods of taking RGB images as input need to learn with geometric constraints and ignore the depth information in the picture, leading to the method being too complicated and inefficient. Although some image-based methods use depth map information for post-calibration and correction, such methods usually require a high-precision depth estimation network. The methods of using the pseudo radar point cloud as input easily introduce noise in the conversion process of depth information to the pseudo radar point cloud, which cause a large deviation in the detection process and ignores semantic information simultaneously. We introduce dynamic convolution guided by the depth map into the feature extraction network, the convolution kernel of dynamic convolution automatically learns from the depth map of the image. It solves the problem that depth information and semantic information cannot be used simultaneously and improves the accuracy of monocular 3D object detection. MonoDCN is able to significantly improve the performance of both monocular 3D object detection and Bird’s Eye View tasks within the KITTI urban autonomous driving dataset.

## Introduction

3D object detection is an indispensable prerequisite for machines to perceive the physical environment and has been widely used in autonomous driving and robot navigation. 3D object detection methods can be divided into two categories according to the implementation cost. One is to use LIDAR sensors [1–4] to obtain 3D point cloud information or to use stereo cameras [5–8] for stereo depth estimation. The LiDAR sensors and stereo cameras used in this method are expensive, making the threshold for 3D object detection too high; the other is a low-cost monocular 3D object detection method. This kind of method has received extensive attention from related industries.

The existing monocular 3D object detection methods are mainly divided into two categories, namely image-based methods [5, 9–19] and pseudo-LiDAR-based methods [20–23]. Image-based methods generally learn through geometric constraints between 2D and 3D, including object shape information, ground information, key points, etc., and use these data to constrain 3D detection in the loss function to learn better. The pseudo-LiDAR-based methods based on first predict the depth map of an input image using an external monocular depth estimator, then predict the distance of objects from the estimated depth map using a point cloud-based 3D object algorithm.

Most image-based monocular 3D object detection methods need to learn from geometric constraints and ignore the picture’s depth information, resulting in the method’s complexity and low efficiency. The pseudo-LiDAR-based methods use the estimated depth map to project the 2D image information into the 3D space to complete the conversion of the pseudo point cloud. There may be deviations during the conversion process. At the same time, this method ignores the semantic information in the image, causing the final result to be biased.

Most prior methods cannot use the semantic information in the image and the depth information in the depth map simultaneously. Some image-based methods use depth map information for post-calibration and correction, but this method usually requires a high-precision depth estimation network. To solve this problem, we introduce dynamic convolution on the image-based method and do not convert the predicted depth map into a pseudo point cloud. In the case of 3D object detection, a smoother depth map and dynamic convolution network are used. We use the depth map generated by the densedepth network [24] as a guide for the dynamic convolution layer in the feature extraction network. The convolution kernel in the dynamic convolution network is learned and generated from the depth map, and applied locally to each pixel and channel of a single image sample, so that different images use different convolution kernels for feature extraction instead of learning the global kernel to apply to all images. The information extracted from the depth map essentially bridges the gap between the image representation and the 3D point cloud representation. At the same time, data enhancement is used for the input image, and the red and green channels in the input channel RGB are interchanged, which can improve performance and calculation efficiency.

To summarize, our contributions are the following:

- We formulate a monocular 3D object detection based on the dynamic convolution network (MonoDCN). The convolution kernel of dynamic convolution is generated by depth map information, and the dynamic convolution is used to replace the 2D convolution in the feature extraction network.
- Our method directly predicts 3D bounding boxes from RGB images with a compact architecture, making the training and inference simple and efficient.
- We evaluate the proposed module and design methods on the monocular 3D Object Detection and Bird’s Eye View tasks of the KITTI dataset, and we achieve competitive results.

## Related works

### Image-based monocular 3D detection

Monocular image is naturally of limited 3D information compared with LiDAR or stereo vision. Therefore, most of the current image-based methods [5, 9–19] improve the detection effect with the help of prior knowledge or geometric constraints and other auxiliary information. Specifically, [9, 11, 15, 18, 19] improve the representation ability of the models by introducing new geometric constraints. Mono3D [9] uses semantic segmentation and context prior knowledge to generate 3D candidate clusters. MonoGRNet [11] uses sparse supervision to directly predict the depth of the center of the object’s 3D bounding box. By estimating the position in the horizontal and vertical dimensions, 3D positioning is further realized. MonoRCNN [15] proposes a novel geometry-based distance decomposition to recover the distance by its factors. MonoPair [18] propose a novel method to improve the monocular 3D object detection by considering the relationship of paired samples. [19] proposed an IoU-oriented loss for 3D size estimation. [13, 16, 17] take 3D detection as the key point detection task, and use more detailed key point annotation position, position information between key points, etc. as the training label. M3D-RPN [12] proposes deep space-aware convolution, which uses unshared kernels in row space to learn space-aware features. However, this rough and fixed spatial division has deviations and cannot capture the scale and local structure of the object. In addition, [10, 14] improve the depth estimation algorithm to make the estimated depth maps more accurate, and use them to enhance the input RGB image or use the depth map information for post-calibration and correction. However, the generalization of these methods is bounded by that of the monocular depth estimation network.

### Pseudo LIDAR-based monocular 3D detection

Weng and Kitani firstly proposed the concept of the pseudo point cloud in [22], but the network structure and overall performance need to be improved. MV3D [20] fused RGB image and pseudo point cloud data as the input of the model, using these fused features to predict the object category, and at the same time return to a fixed direction 3D box, but the calculation process is more cumbersome. AM3D [21] firstly obtains the depth information and the 2D object position prior, then maps the 2D depth information to the 3D space and performs subsequent processing in the form of point cloud data. DA-3Ddet [23] leverages the domain adaptation approach to customize the features from pseudo-LiDAR domain to real-LiDAR domain, to bridge the performance gap between monocular-based and LiDAR point-based 3D detection methods. The effect is effectively improved, but the process from 2D information to 3D point cloud information may introduce some error information, leading to incorrect judgments in the subsequent detection process.

### Depth estimation

People pay more and more attention to the development of monocular depth estimation. In recent years, although many pixel-level depth estimation networks [24, 25] have been proposed, these methods are not suitable for 3D object localization. When performing pixel-level depth regression, the loss function weighs each depth map pixel and treats them indiscriminately. Normally, the loss value of each pixel is finally added up and optimized together. However, in reality, there are far fewer pixels in the object than in the background. Therefore, a lower average error does not mean that the depth value of the pixel in the detection object is accurate. In addition, dense depth estimation often comes from the disparity map, which may cause larger errors when detecting distant areas, greatly reducing the performance of 3D positioning.

## MonoDCN

**Fig 1:**
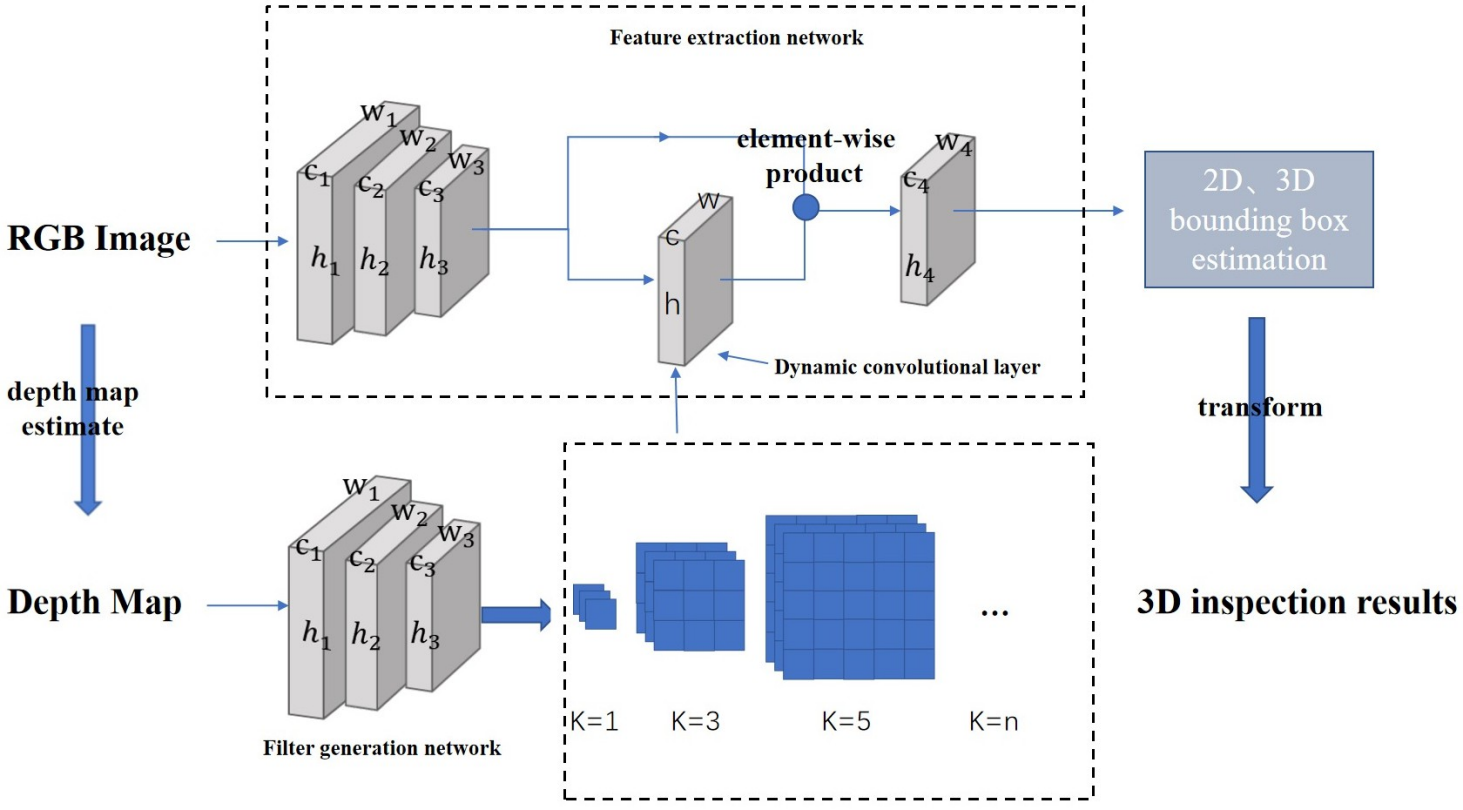
MonoDCN for 3D object detection from a monocular RGB image. Overall framework consists of three important components: a depth map generation network, a feature extraction network, and a dynamic convolution layer guided by the depth map.

Our overall framework consists of three important components: a depth map generation network, a feature extraction network, and a dynamic convolution layer guided by the depth map. We refer to our method as monocular 3D object detection based on the dynamic convolution network (MonoDCN), as illustrated in Fig 1. To make full use of the information in the depth map and RGB image, our backbone is represented as a two-branch network: the first branch is the feature extraction network of RGB images, and the other branch is the filter generation network, generated for dynamic convolutional layers convolution kernel. These two networks take RGB image and depth map as input, respectively, and then use the feature map of a feature extraction network as the input of 2D and 3D detection heads to obtain the position information of the detection object, and finally adopt non-maximum suppression and data conversion for visualization.

### Backbone

#### Feature extraction network

The backbone of the feature extraction network for RGB images is ResNet-50 [26]. ResNet-50 is pre-trained on the ImageNet [27] classification dataset without using its final FC and pooling layer. In order to obtain a larger field of view, set the network stride to 16. At the same time, in order to make better use of the correlation between the depth map and the RGB image, we inserted a dynamic convolution layer in the feature network of the RGB image. It is introduced in detail in the following section.

#### Depthmap generation network

**Fig 2:**
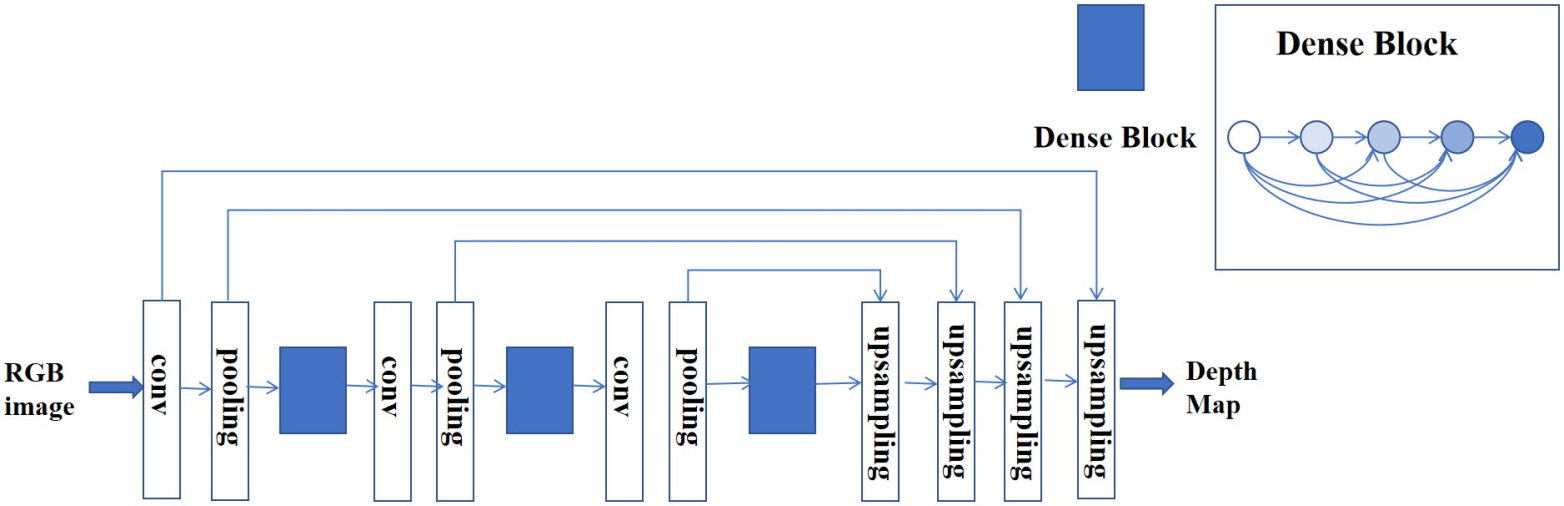
Encoder decoder network structure. We employ a straightforward encoder-decoder architecture with skip connections. The encoder part is a pre-trained truncated DenseNet-169 [21] with no additional modifications. The decoder is composed of basic blocks of convolutional layers.

The depth map generation network is based on densedepth network [24], which is a typical encoder and decoder network, using DenseNet-169 [28] with a tighter network connection mechanism as the backbone. The structure of the depth map generation network is shown in Fig 2. DenseNet-169 has the advantages of a narrower network and fewer parameters. The focus of the network structure is dense connection. Each layer has direct access to the gradients from the loss function and the original input signal, to reduce overfitting. DenseNet-169 is pre-processed on ImageNet [27]. The encoder encodes the input RGB image into a feature vector. Then the feature vector is sent to a series of continuous up-sampling layers to construct the final depth map with a series of extracted features. These up-sampling layers and their related skip connections constitute the densedepth decoder. The obtained depth map is used as the input of the filter generation network to generate the convolution kernel of the dynamic convolution layer.

### Dynamic convolution

Dynamic convolution is the core of our network. The convolution kernel of dynamic convolution is trained and generated by the depth map, using the feature information in the depth map as a guide. We firstly introduce the concept of dynamic convolution. Generally speaking, the convolution kernel in dynamic convolution is a form of dynamic filter, in which the convolution kernel changes with the input picture. Since the convolution kernel generated based on the depth map is actually a feature body, a simple way to do this is to convert the feature body to a specific convolution kernel of the *h*_*n*_×*w*_*n*_ input map, and then input the convolution kernel into the dynamic convolution layer, where *h*_*n*_ and *w*_*n*_ are the height and width of the feature maps at layer n, *c*_*n*_ is the number of channels of the feature map is generally RGB three channels, k represents the size of the convolution kernel of the dynamic convolution layer. The convolution kernel in the dynamic convolution layer is generated by the filter generation network. For the filter generation network, the backbone also uses the first three blocks of ResNet-50 for calculation, which is the same as the first three layers of the feature extraction network. Structure reuse to reduce computing costs. The number of channels in each block in the first three layers of the feature extraction network and the filter generation network is also the same. The main task of this network is to learn the depth information in the depth map to obtain a dynamic filter (convolution kernel in the dynamic convolution), and apply it to the dynamic convolution layer of RGB image feature extraction. The feature maps obtained by the first three layers of the feature extraction network and the feature maps obtained by the dynamic convolution layer are subjected to the element-wise product operation and used as the input of the last layer of the feature extraction network.

**Fig 3:**
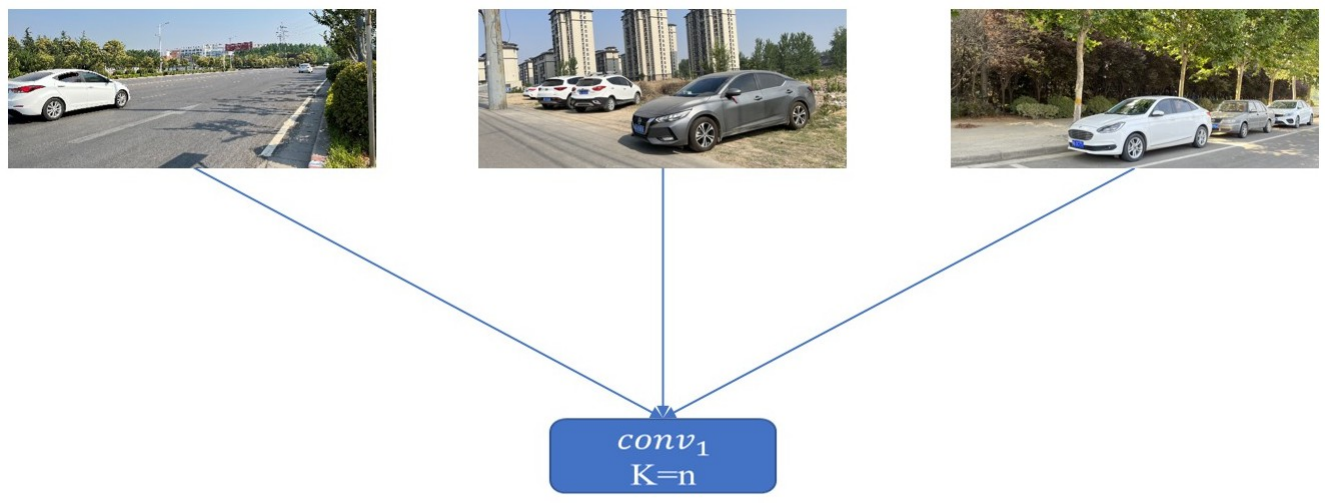
Traditional 2D convolution. The input image has no effect on the characteristics of the convolution layer, n is a fixed value.

**Fig 4:**
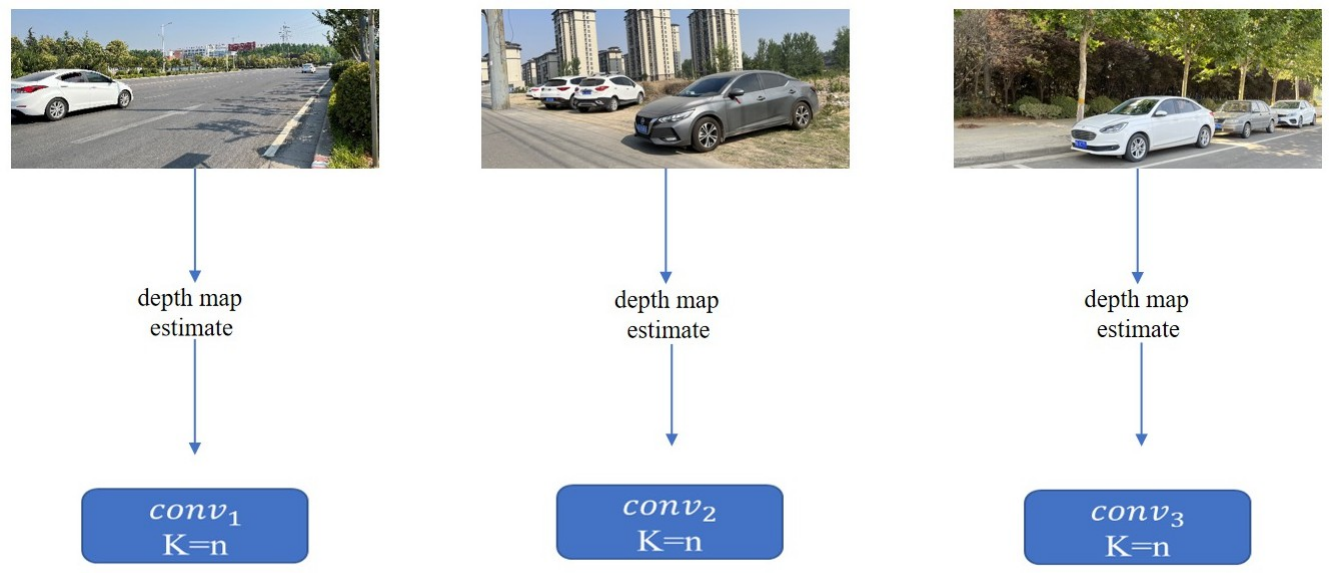
Dynamic convolution. The convolution kernel of dynamic convolution changes according to the input image, n is variable.

The traditional 2D full convolution uses a fixed convolution kernel for feature extraction. Different images can only use the same convolution kernel when performing feature extraction, resulting in poor training effects. It cannot effectively reflect the depth-related scale changes of the object, nor can it effectively reflect the spatial relationship between foreground and background pixels. On the other hand, the pseudo-LiDAR-based methods rely too much on depth accuracy and ignore semantic information in RGB images. To solve these problems at the same time, we introduce dynamic convolution [29]. The convolution kernel of the dynamic convolution layer is different for different images, and it is sample-specific. The comparison of traditional 2D full convolution and dynamic convolution is shown in Figs 3 and 4. The convolution kernel of dynamic convolution changes according to the input image, but for the traditional 2D convolution, the input image has no effect on the convolution layer.

### 3D box prediction

In this work, we adopt a single-stage detector based on a priori-based 2D-3D anchor box [30]. Firstly, the feature map $I_{4}\in R^{h_{4}\times w_{4}}$ output by the feature extraction network is used as the detector input, and the 2D and 3D frame coordinate values are predicted. Then the 2D and 3D frame regression are completed by non-maximum suppression and data transformation.

#### Formulation

*Inputs*. The output feature map $I_{4}\in R^{h_{4}\times w_{4}}$ of our backbone network with a network stride factor of 16. Following common practice, we use a calibrated setting which assumes that per image camera intrinsic *δ* ∈ *R*^3×4^ are available both at the train and test time. The 3D-to-2D projection can be written as Eq (1):

$$\begin{array}{c} {\left[\begin{array}{c} x\\ y\\ 1 \end{array}\right]}_{p}•z_{3D}=\delta •{\left[\begin{array}{c} x\\ y\\ z\\ 1 \end{array}\right]}_{3D} \end{array}$$ (1)

Where [*x*, *y*, *z*]_3*D*_ denotes the horizontal position, height and depth of the 3D point in camera coordinates, and [*x*, *y*]_*p*_ is the projection of the 3D point in 2D image coordinates, *z*_3*D*_ represents the depth of its true label.

**Fig 5:**
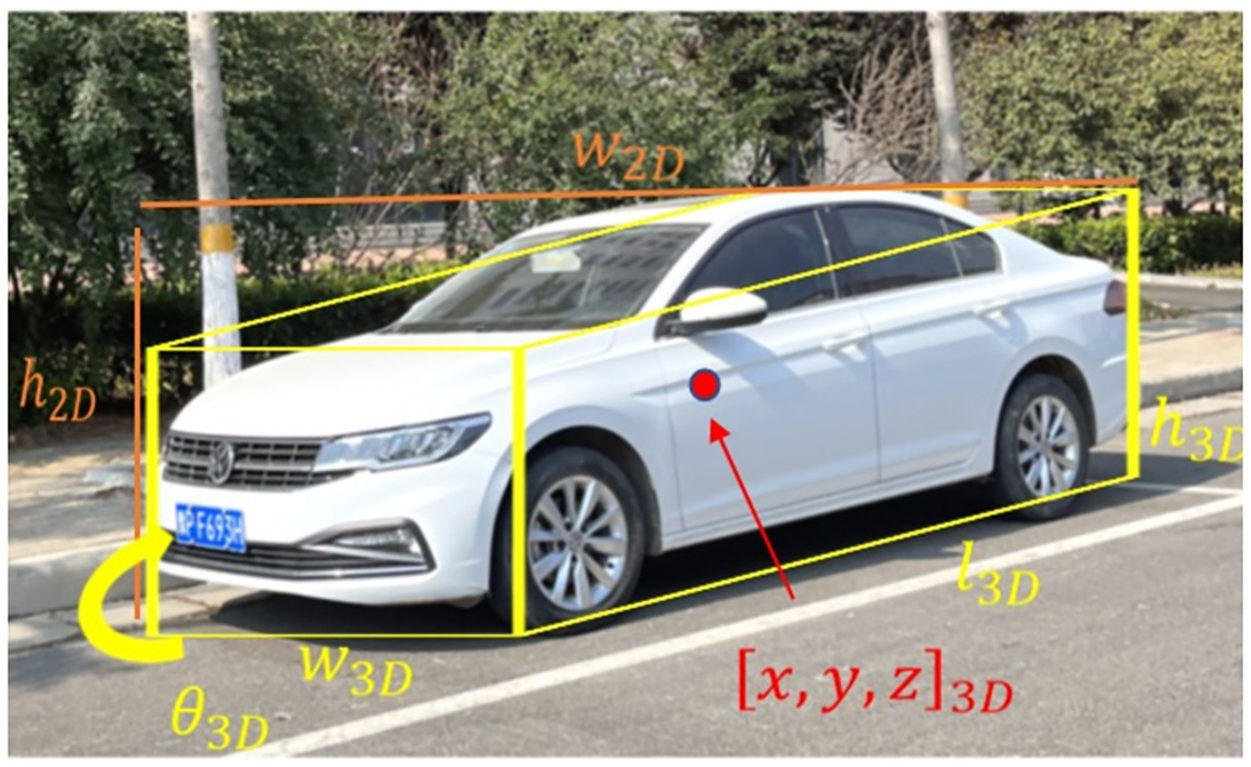
Anchor formulation. We depict each parameter in the 2D/3D anchor formulation.

**Fig 6:**
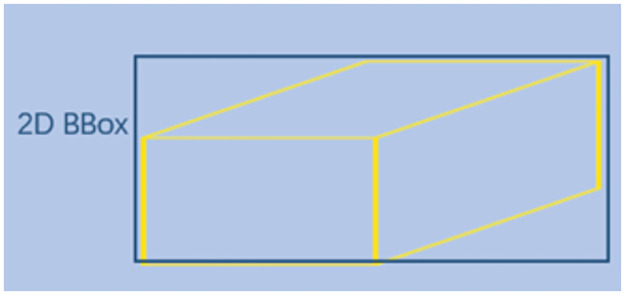
2D bounding box representation. We depict the smallest bounding rectangle of the projected 3D box as the 2D bounding box of our true label.

*Ground truth*. We define a ground truth (GT) box using the following parameters:[*x*, *y*, *w*, *h*]_2*D*_ represents the 2D bounding box, where (x, y) is the center of the 2D box, w, h are the width and height of the 2D box; [*x*, *y*, *z*]_3*D*_ represents the position of the 3D center in camera coordinates; [*w*, *h*, *l*]_3*D*_(the height, width, and length(in meters) of the 3D object), and the different-center posture in the 3D space *θ*_3*D*_(the object’s observation angle, the range is [−*π*, *π*]) [31]. We use the smallest bounding rectangle of the projected 3D box as the 2D bounding box for our ground-truth label, as shown in Figs 5 and 6.

*Outputs*. We choose *n*_*α*_ to represent the number of anchor boxes, and *n*_*c*_ to represent the number of classes. For each input position (i, j), the corresponding output anchor box contains 35+*n*_*c*_ parameters: $\left\{{\left[{\;t}_{x},{\;t}_{y},{\;t}_{w},{\;t}_{h}\right]}_{2D},{\left[{\;t}_{x},{\;t}_{y}\right]}_{p},{\left[{\;t}_{z},{\;t}_{w},{\;t}_{h},{\;t}_{l},{\;t}_{\alpha }\right]}_{3D},t_{c}^{\left(m\right)},s\right\}$, where [*t*_*x*_, *t*_*y*_, *t*_*w*_, *t*_*h*_]_2*D*_ represents the predicted 2D box, and [*t*_*x*_, *t*_*y*_]_*p*_ is the predicted 3D center in 2D projection position, [*t*_*z*_, *t*_*w*_, *t*_*h*_, *t*_*l*_, *t*_*α*_]_3*D*_ represents the predicted depth, 3D shape; and rotation; $t_{c}^{\left(m\right)}=\{{\left[t_{x}^{\left(m\right)},t_{y}^{\left(m\right)}\right]}_{p},{\left[t_{z}^{\left(m\right)}\right]}_{3D}\}$(*m* ∈ {1, 2, …, 8}), which represents the 8 corner points of the 3D box, s is the confidence score of each class, the input size is *h*_4_×*w*_4_×*n*_*α*_×(35+ *n*_*c*_), where (*h*_4_, *w*_4_) is the size of the input image, and the down-sampling factor is 16. The output is actually based on the anchor transformation of the 2D-3D box.

*Anchor*. As in [12] and [30], firstly define the 2D-3D anchor point in the 2D space, and then use the corresponding relationship in the training data set to calculate its part in the three-dimensional space. Use two space parameters to define a template anchor: [*A*_*x*_, *A*_*y*_, *A*_*w*_, *A*_*h*_]_2*D*_, [*A*_*z*_, *A*_*w*_, *A*_*h*_, *A*_*l*_, *A*_*α*_]_3*D*_, where [*A*_*z*_, *A*_*w*_, *A*_*h*_, *A*_*l*_, *A*_*α*_]_3*D*_ represents a 3D anchor(depth, shape, rotation), and [*A*_*x*_, *A*_*y*_, *A*_*w*_, *A*_*h*_]_2*D*_ represents a 2D anchor. For each 2D anchor, we use the statistical data of all 3D boxes that match the real label as its corresponding 3D anchor [*A*_*z*_, *A*_*w*_, *A*_*h*_, *A*_*l*_, *A*_*α*_]_3*D*_. Note that we use the same anchor parameter [*A*_*x*_, *A*_*y*_]_2*D*_ for regressions of [*t*_*x*_, *t*_*y*_]_2*D*_ and [*t*_*x*_, *t*_*y*_]_*p*_. The anchor-based method enables our network to learn the relative value (residual) of the true label, which significantly reduces the difficulty of learning.

Finally, we combine the network output with a predefined anchor to obtain an estimated 3D box. The specific combination method is as Eqs (2)–(7):

$$\begin{array}{c} {\left[x^{{}^{\prime }},y^{{}^{\prime }}\right]}_{2D}={\left[{\;A}_{x},A_{y}\right]}_{2D}+{\left[{\;t}_{x},{\;t}_{y}\right]}_{2D}*{\left[{\;A}_{w},{\;A}_{h}\right]}_{2D} \end{array}$$ (2)

$$\begin{array}{c} {\left[x^{{}^{\prime }},y^{{}^{\prime }}\right]}_{p}={\left[{\;A}_{x},A_{y}\right]}_{2D}+{\left[{\;t}_{x},{\;t}_{y}\right]}_{p}*{\left[{\;A}_{w},{\;A}_{h}\right]}_{2D} \end{array}$$ (3)

$$\begin{array}{c} {\left[x^{{}^{\prime }\left(m\right)},y^{{}^{\prime }\left(m\right)}\right]}_{p}={\left[{\;A}_{x},A_{y}\right]}_{2D}+{\left[t_{x}^{\left(m\right)},t_{y}^{\left(m\right)}\right]}_{p}*{\left[{\;A}_{w},{\;A}_{h}\right]}_{2D} \end{array}$$ (4)

$$\begin{array}{c} {\left[w^{{}^{\prime }},h^{{}^{\prime }}\right]}_{2D}={\left[{\;A}_{w},A_{h}\right]}_{2D}•\text{exp}({\left[{\;t}_{w},{\;t}_{h}\right]}_{2D}) \end{array}$$ (5)

$$\begin{array}{c} {\left[w^{{}^{\prime }},h^{{}^{\prime }},l^{{}^{\prime }}\right]}_{3D}={\left[{\;A}_{w},A_{h},A_{l}\right]}_{3D}•\text{exp}({\left[{\;t}_{w},{\;t}_{h},t_{l}\right]}_{3D}) \end{array}$$ (6)

$$\begin{array}{c} {\left[z^{{}^{\prime }},z^{{}^{\prime }\left(m\right)},\alpha^{{}^{\prime }}\right]}_{p}=\left[A_{z},A_{z},A_{\alpha }\right]+{\left[t^{z},t^{z},t_{\alpha }\right]}_{3D} \end{array}$$ (7)

[*x*′, *y*′]_*p*_ represents the projection coordinates of the predicted 3D center on 2D, and [*z*′, *z*′^(*m*)^, *α*′]_*p*_ represents the depth value and rotation angle of the center of the predicted 3D frame and its eight vertices.

#### Loss functions

In the object detection task, the ratio of the number of front sights to the background points is unbalanced, and there are many irrelevant background points. The ordinary loss function cannot tap the importance of the front sights. Therefore, we adopt the Focal Loss [32] as the total Loss function, which can reduce the weight of many background points in training so that the training pays more attention to the object. The overall loss function includes object classification loss, 2D regression loss, 3D regression loss, and corner loss. The overall loss function is as Eq (8):

$$\begin{array}{c} L={\left(1-s_{c}\right)}^{\gamma }(L_{class}+L_{2D}+L_{3D}+L_{corner}) \end{array}$$ (8)

The above formula, *L*_*class*_, *L*_2*D*_, *L*_3*D*_, *L*_*concer*_ represents the object classification loss, 2D regression loss, 3D regression loss, and corner loss, *s*_*c*_ is the classification score of the object class, and *γ* is the focus parameter, which can adjust the rate at which the sample weight decreases; during the experiment, *γ* take 2.

The object classification work uses the standard cross-entropy loss as Eq (9):

$$\begin{array}{c} L_{class}=-log(s_{c}) \end{array}$$ (9)

The SmoothL1 regression loss is selected for 2D and 3D regression, and each specific loss function is defined as Eqs (10)–(12):

$$\begin{array}{c} L_{2D}=SmoothL1({\left[x^{{}^{\prime }},y^{{}^{\prime }},w^{{}^{\prime }},h^{{}^{\prime }}\right]}_{2D},{\left[x,y,w,h\right]}_{2D}) \end{array}$$ (10)

$$\begin{array}{c} L_{3D}=SmoothL1({\left[w^{{}^{\prime }},h^{{}^{\prime }},l^{{}^{\prime }},z^{{}^{\prime }},\alpha^{{}^{\prime }}\right]}_{3D},{\left[w,h,l,z,\alpha \right]}_{3D})+SmoothL1({\left[x^{{}^{\prime }},y^{{}^{\prime }}\right]}_{p},{\left[x,y\right]}_{p}) \end{array}$$ (11)

$$\begin{array}{c} L_{corner}=\frac{1}{8}\sum SmoothL1({\left[x^{{}^{\prime }\left[m\right]},y^{{}^{\prime }\left[m\right]}\right]}_{p},{\left[x^{\left[m\right]},y^{\left[m\right]}\right]}_{p})+\;SmoothL1({\left[z^{{}^{\prime }\left(m\right)}\right]}_{3D},{\left[z\right]}_{3D}) \end{array}$$ (12)

Where [*x*^(*m*)^, *y*^(*m*)^]_*p*_ is the eight vertices of the 3D box, and [*z*]_3*D*_ is the corresponding depth.

## Experiments

We evaluate our proposed framework on the KITTI [33] 3D object detection dataset under two core 3D localization tasks: Bird’s Eye View (BEV) and 3D Object Detection. We evaluate our method on split1 [5] on the KITTI dataset and analyze the key components that make up our framework. We further visualize qualitative examples of MonoDCN for multiple types of 3D object detection in different driving scenarios.

### KITTI

The KITTI dataset of 3D object detection consists of 7481 training images and 7518 test images, as well as corresponding point clouds and calibration parameters. It includes 80,256 2D and 3D labeled three types of objects, namely cars, pedestrians, and bicycles. According to the occlusion and truncation level of the object, each 3D ground truth frame is divided into three difficulty categories: easy, moderate, and difficult. The KITTI dataset has two kinds of the training set and validation set segmentation, one is split1 [5], which consists of 3712 training images and 3769 verification images, and the other is split2 [34], which consists of 3682 training images and 3799 verification images. The dataset is used to perform three tasks: 2D detection, 3D detection, and Bird’s Eye View.

We evaluate our model on the KITTI [33] dataset. This paper focuses on the training and validation of 3D detection and Bird’s Eye View under split1. The specific experimental configuration is shown in Table 1.

**Table 1:** Experimental configuration.

Item	CPU	Computing memory	GPU	System	CUDA
Content	E5-2678 v3	16GB	NVIDIA RTX 2080 Ti	Ubuntu 18.04	CUDA 10.2

#### Implementation details

In order to facilitate subsequent experiments, the input images are firstly scaled to 512 × 1760, Which are used for feature extraction and depth map generation respectively. According to the generated depth map training, dynamic convolution is obtained. Then, the feature map generated by the first three layers of the feature extraction network and the feature map generated by the dynamic convolution layer are multiplied by the corresponding elements, and the obtained result is used as the input of the last layer of the feature extraction network, and the 2D and 3D frame coordinates of the target are finally output. After non-maximum suppression, the final visualization result is output. The experiment is evaluated under the IoU threshold of 0.5 and 0.7, the learning rate is selected as 0.01, which decays with training, the optimizer selects the SAD algorithm, the momentum is selected as 0.8, and the batch size is 8, and the training is performed for 500 rounds.

#### Experimental results

**Fig 7:**
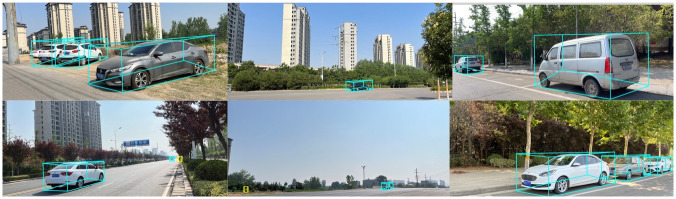
Qualitative examples. We visualize qualitative examples of our multi-class 3D object detection method. All illustration images are taken manually by us and used as the final detection effect demonstration.

The visualized 3D object detection result is shown in Fig 7. The figure only shows the estimated bounding box, excluding the true value and its category. The performance comparison between our method and the current mainstream monocular 3D object detection methods is shown in Tables 2 and 3. Among them, AP represents the average precision, *AP*_3*D*_ represents the average accuracy of the 3D detection frame, and *AP*_*BEV*_ represents the average accuracy of the detection frame in the BEV view, which is calculated using the $AP{|}_{R_{11}}$ standard.

**Table 2:** Bird’s eye view.

Method	AP^BEV^(IoU ≥ 0.7)			AP^BEV^(IoU ≥ 0.5)		
	Easy	Mod	Hard	Easy	Mod	Hard
MoNo3D [9]	5.22	5.19	4.13	30.50	22.39	19.16
MF3D [10]	22.03	13.63	11.60	55.02	36.73	31.27
MoNoGRNet [11]	24.97	19.44	16.30	54.21	39.69	33.06
M3D-RPN [12]	25.94	21.18	17.90	55.37	42.49	35.29
Ours	30.91	23.51	19.16	59.43	45.51	37.21

Comparison of our method to image-only 3D detection frameworks with the same KITTI validation set on the Bird’s Eye View task (*AP*_*BEV*_).

**Table 3:** 3D detection.

Method	AP^3D^(IoU ≥ 0.7)			AP^3D^(IoU ≥ 0.5)		
	Easy	Mod	Hard	Easy	Mod	Hard
MoNo3D [9]	2.53	2.31	2.31	25.19	18.20	15.22
MF3D [10]	10.53	5.69	5.39	47.88	29.48	26.44
MoNoGRNet [11]	13.88	10.19	7.62	50.51	36.97	30.82
M3D-RPN [12]	20.27	17.06	15.21	48.96	39.57	33.01
Ours	21.92	17.68	15.27	50.61	41.86	34.57

Comparison of our method to image-only 3D detection frameworks with the same KITTI validation set on the 3D Detection task (*AP*_3*D*_).

### Ablations

In order to verify the degree of influence of each processing part on the network of this article, we conduct an ablation study on our model, only a comparative experiment was carried out under 3D detection, and the results are shown in Table 4. It can be seen from the Table 4 that data enhancement and dynamic convolution processing can effectively improve the AP value of 3D object detection. The densedepth network is slightly helpful for the model, because the densedepth network in the model is used in combination with dynamic convolution, and the densedepth network provides information for dynamic convolution.

**Table 4:** Ablations.

Part			AP/%		
Densedepth	Data Augmentation	Dynamic convolution	Easy	Mod	Hard
-	-	-	19.24	16.25	13.67
√	-	-	19.63	16.71	13.59
-	√	-	19.80	17.03	14.31
-	-	√	20.43	17.36	14.72
√	√	-	19.96	17.09	14.42
√	-	√	20.83	17.54	14.98
-	√	√	20.64	17.49	15.01
√	√	√	21.92	17.68	15.27

We list the influence of densedepth, Data Augmentation and Dynamic convolution on the model.

#### Dynamic convolution

We use dynamic convolution to improve the model’s perception of depth information to improve the accuracy of 3D object detection. The network can learn specific kernels applied to different pictures. To better understand the effect of dynamic convolution, we conduct experiments and use the same experimental configuration. The network model uses dynamic convolution. The traditional 2D convolution is replaced, and the rest is unchanged. The results obtained are shown in Table 4.

## Conclusion

We use a dynamic convolution network based on depth map features for monocular 3D object detection. Our method has an excellent performance in three-dimensional detection, positioning, and pose estimation among the existing monocular methods. This paper proposes to use the depth information in the depth map to generate the convolution kernel of the dynamic convolution network, use dynamic convolution to replace the traditional 2D full convolution, and combine the RGB image information with the depth information, which effectively improves the monocular 3D object detection Accuracy.
